# A Thyroid Hormone-Independent Molecular Fingerprint of 3,5-Diiodothyronine Suggests a Strong Relationship with Coffee Metabolism in Humans

**DOI:** 10.1089/thy.2018.0549

**Published:** 2019-12-16

**Authors:** Maik Pietzner, Josef Köhrle, Ina Lehmphul, Kathrin Budde, Gabi Kastenmüller, Georg Brabant, Henry Völzke, Anna Artati, Jerzy Adamski, Uwe Völker, Matthias Nauck, Nele Friedrich, Georg Homuth

**Affiliations:** ^1^ Institute of Clinical Chemistry and Laboratory Medicine, University Medicine Greifswald, Greifswald, Germany.; ^2^ DZHK (German Center for Cardiovascular Research), Partner Site Greifswald, Greifswald, Germany.; ^3^ MRC Epidemiology Unit, Institute of Metabolic Science, University of Cambridge, Cambridge, United Kingdom.; ^4^ Institut für Experimentelle Endokrinologie, Charité-Universitätsmedizin Berlin, Corporate Member of Freie Universität Berlin, Humboldt-Universität zu Berlin, and Berlin Institute of Health, Berlin, Germany.; ^5^ Institute of Bioinformatics and Systems Biology, Helmholtz Zentrum München, Neuherberg, Germany.; ^6^ Medical Clinic I, University of Lübeck, Lübeck, Germany.; ^7^ DZD (German Center for Diabetes Research), Site Greifswald, Greifswald, Germany.; ^8^ Institute for Community Medicine, University Medicine Greifswald, Greifswald, Germany.; ^9^ Research Unit of Experimental Genetics, Genome Analysis Center, Molecular Endocrinology and Metabolism, Helmholtz Zentrum München, Neuherberg, Germany.; ^10^ Lehrstuhl für Experimentelle Genetik, Technische Universität München, Freising-Weihenstephan, Germany.; ^11^ DZD (German Center for Diabetes Research), München-Neuherberg, Germany.; ^12^ Department of Biochemistry, Yong Loo Lin School of Medicine, National University of Singapore, Singapore, Singapore.; ^13^ Department of Functional Genomics, Interfaculty Institute for Genetics and Functional Genomics, University Medicine Greifswald, Greifswald, Germany.

**Keywords:** 3,5-diiodothyronine, metabolomics, caffeine metabolism, thyroid, thyrotoxicosis

## Abstract

***Background:*** In numerous studies based predominantly on rodent models, administration of 3,5-diiodo-L-thyronine (3,5-T2), a metabolite of the thyroid hormones (TH) thyroxine (T4) and triiodo-L-thyronine (T3), was reported to cause beneficial health effects, including reversal of steatohepatosis and prevention of insulin resistance, in most instances without adverse thyrotoxic side effects. However, the empirical evidence concerning the physiological relevance of endogenously produced 3,5-T2 in humans is comparatively poor. Therefore, to improve the understanding of 3,5-T2-related metabolic processes, we performed a comprehensive metabolomic study relating serum 3,5-T2 concentrations to plasma and urine metabolite levels within a large general population sample.

***Methods:*** Serum 3,5-T2 concentrations were determined for 856 participants of the population-based Study of Health in Pomerania-TREND (SHIP-TREND). Plasma and urine metabolome data were generated using mass spectrometry and nuclear magnetic resonance spectroscopy, allowing quantification of 613 and 578 metabolites in plasma and urine, respectively. To detect thyroid function-independent significant 3,5-T2—metabolite associations, linear regression analyses controlling for major confounders, including thyrotropin and free T4, were performed. The same analyses were carried out using a sample of 16 male healthy volunteers treated for 8 weeks with 250 μg/day levothyroxine to induce thyrotoxicosis.

***Results:*** The specific molecular fingerprint of 3,5-T2 comprised 15 and 73 significantly associated metabolites in plasma and urine, respectively. Serum 3,5-T2 concentrations were neither associated with classical thyroid function parameters nor altered during experimental thyrotoxicosis. Strikingly, many metabolites related to coffee metabolism, including caffeine and paraxanthine, formed the clearest positively associated molecular signature. Importantly, these associations were replicated in the experimental human thyrotoxicosis model.

***Conclusion:*** The molecular fingerprint of 3,5-T2 demonstrates a clear and strong positive association of the serum levels of this TH metabolite with plasma levels of compounds indicating coffee consumption, therefore pointing to the liver as an organ, the metabolism of which is strongly affected by coffee. Furthermore, 3,5-T2 serum concentrations were found not to be directly TH dependent. Considering the beneficial health effects of 3,5-T2 administration observed in animal models and those of coffee consumption demonstrated in large epidemiological studies, one might speculate that coffee-stimulated hepatic 3,5-T2 production or accumulation represents an important molecular link in this connection.

## Introduction

The classical thyroid hormones (TH) thyroxine (T4) and triiodothyronine (T3) represent key regulators of development and metabolic homeostasis (1). In addition, within the last two decades, considerable research efforts (2) resulted in an expansion of the spectrum of metabolically active TH metabolites, including 3-iodothyronamine (3) and 3,5-diiodo-L-thyronine (3,5-T2) (4). In hypothyroid rodent models, 3,5-T2 was described to exert numerous beneficial metabolic effects (4). Under high-fat diet conditions, administration of 3,5-T2 prevented weight gain (5) and the development of high-fat diet induced insulin resistance (6,7) or even reversed hepatic steatosis (8–11).

Strikingly, it was reported that 3,5-T2 exerts these effects independent of classical TH-signaling through nuclear receptors and hence without the well-known thyrotoxic side effects on bone, heart, and the hypothalamus/pituitary/thyroid (HPT) axis. Consequently, 3,5-T2 was considered a promising drug for the treatment of obesity and metabolic syndrome. However, in particular, more recent animal studies (12,13) challenged this optimistic view by demonstrating adverse consequences of dose-dependent 3,5-T2 treatment such as an increased heart weight and suppression of the HPT-axis (14–18). This confirmed the results of earlier *in vivo* studies in rats that indicated differential liver stimulating- and thyrotropin (TSH)-suppressing thyromimetic effects of 3,5-T2 compared with T3 (19).

Apart from these conflicting results from animal studies, data on 3,5-T2 in humans are generally sparse. No convincing translation of findings from animal models to humans has been presented yet. Administration of the 3,5-T2 synthetic analogue TRC150094 at a dose of 50 mg/day for 4 weeks failed to improve lipid and glucose metabolic homeostasis in volunteers presenting with an elevated cardiometabolic risk (20). Studies in the 1980s reported an age-dependent increase as well as sex-specific differences in serum 3,5-T2 concentrations [3,5-T2], but no consistent alterations in thyroid disease (21–26).

However, only recently the development of a novel immunoassay based on a monoclonal antibody against 3,5-T2 (27) enabled the precise and reliable determination of [3,5-T2] in humans. Using this assay, no alterations in endogenous [3,5-T2] with respect to the thyroid state in patient cohorts were found (27), and even a population-based approach (28) comprising more than 900 subjects revealed only moderate associations with TSH and plasma glucose concentrations, while the predicted inverse associations with blood lipid or anthropometric parameters were not detected.

More detailed phenotyping of the same cohort was performed by small-molecule content profiling of the urine samples using proton nuclear resonance (^1^H-NMR) spectroscopy. This urine metabolomic approach revealed 3,5-T2-related alterations with respect to fatty acid oxidation or oxidative stress that might provide links to observations in animal models mentioned above (29). Strikingly, a strong positive association of [3,5-T2] with urinary excretion of trigonelline raised particular interest as similar beneficial effects on glucose metabolism were described for both compounds (6,30–32).

To follow up on this notable observation, and to characterize additional metabolic implications of 3,5-T2 among humans, we substantially extended our previous ^1^H-NMR-study by reanalyzing the same population-based cohort this time using nontargeted as well as targeted metabolomic approaches and plasma samples in addition to urine. Serum TH measurements that became available for the entire cohort allowed us to test for TH-independent effects of 3,5-T2.

## Materials and Methods

### Study population

The Study of Health in Pomerania-TREND (SHIP-TREND) is a population-based study recruited in Western Pomerania, a rural region in northeastern Germany. A detailed description of the study population and sampling procedure can be found elsewhere (33). In total, 4420 invited subjects decided to participate in the study (50.1% response). All participants gave written informed consent before participation. The study was approved by the Ethics Committee of the University of Greifswald and conformed to the principles of the Declaration of Helsinki. SHIP data are publicly available for scientific purposes and can be applied for at www.fvcm.med.uni-greifswald.de/dd_service/data_use_intro.php?lang=ger.

For a subsample of 1000 participants, plasma and urine metabolome data based on mass spectrometry (MS) and NMR were available. Subjects with missing [3,5-T2] values (*n* = 132) or potential confounders (*n* = 3), as well as subjects reporting intake of medication influencing TH concentrations (*n* = 9; amiodarone (ATC code C01BD01) or oral glucocorticoids (ATC code H02AB), were excluded, resulting in a final study sample of 856 subjects. For sensitivity analysis, a euthyroid subpopulation was defined as follows: no intake of thyroid-related medication (*n* = 86; ATC code H03A or H03B) nor a TSH value outside the reference range [*n* = 100; 0.49 mU/L < TSH <3.29 mU/L (34)], thus leaving 690 individuals for the analysis (overlap between criteria existed).

Plasma metabolome data together with determined [3,5-T2] and thyroid function parameters were further available for a human model of experimental thyrotoxicosis, described in detail earlier (35,36). Briefly, 16 healthy male volunteers (ages 22–34 years) were treated with 250 μg levothyroxine (LT4) per day for 8 weeks. Plasma samples were repeatedly collected every four weeks, including a restoration period of eight weeks after completion of the treatment. Quantitative plasma metabolome data were obtained from Metabolon, Inc. (Durham, NC) and included 349 metabolites in total.

### Laboratory measurements and phenotypic characterization

Smoking status (current, former, or never smokers), daily alcohol consumption, and physical activity (≥1 hour training a week) were assessed using computer-aided personal interviews. Waist circumference was measured midway between the lower rib margin and the iliac crest in the horizontal plane. Fasting blood was sampled between 7.00 a.m. and 12.00 p.m. in supine position from the cubital vein. During the same time period, spot urine samples were collected. All samples were either analyzed immediately or stored at −80°C in the Integrated Research Biobank (Liconic, Liechtenstein). Serum concentrations of TSH, free T3 (fT3), and free T4 (fT4) were measured using an immunoassay (Dimension VISTA; Siemens Healthcare Diagnostics, Eschborn, Germany) with functional sensitivities of 0.005 mU/L, 0.77 pmol/L, and 1.3 pmol/L for TSH, fT3, and fT4, respectively. Determination of [3,5-T2] was performed using a monoclonal antibody-based chemiluminescence immunoassay with a functional sensitivity specified as 0.2 nM (27). The interassay variation was between 5.6% and 12.9%. The working range was declared as 0.2 to 10 nM 3,5-T2. More detailed information about the assay has been reported previously (27,28).

### Metabolome analysis

All metabolome measurement techniques are described in detail in the Supplementary Data. Briefly, three different approaches were combined as reported previously (37): (a) nontargeted MS-based profiling of plasma and urine samples; (b) targeted MS-based profiling of plasma samples using the AbsoluteIDQ p180 Kit (BIOCRATES LifeSciences AG, Innsbruck, Austria); and (c) NMR-based profiling of urine samples.

After quality control and preprocessing (Supplementary Data), data on 613 and 587 plasma and urine metabolites, respectively, were available for statistical analyses. Of note, some of these metabolites could not be identified unambiguously by assigning them to a defined chemical structure and thus are subsequently referred with the notation “X” followed by a unique number.

### Statistical analyses

Linear regression models were applied to assess the association between [3,5-T2] (independent variable) and plasma as well as urine metabolites (dependent variable). Since about one-third of the study population exhibited a [3,5-T2] that was below the detection limit of the used assay (0.2 nM), but clearly higher than blank values of the standard curve, subjects were accordingly subdivided into three groups: [3,5-T2] < 0.2 nM (*N* = 316), 0.2 < [3,5-T2] < 0.33 nM (*N* = 261), and [3,5-T2] > 0.33 nM (*N* = 278). Subsequently, linear regression analyses were performed using the defined group variable as exposure. Furthermore, log-transformed [3,5-T2] was used as exposure in the subpopulation with quantified measurements. Models were adjusted for age, sex, waist circumference, blood sampling time, as well as serum TSH and fT4 concentrations. The latter enabled us to control for potential confounding effects of thyroid function on the presented associations.

Associations with TSH, fT3, and fT4 were tested using linear regression following previous work (28), but additionally increasing the sample size from 64 (i.e., participants with conspicuous TSH concentrations) to 856 participants with available [3,5-T2] data. To account for the repeated measurement character of the thyrotoxicosis study, we used linear mixed effects models with [3,5-T2] as fixed effect exposure and plasma metabolites as outcome. Dependency of related samples, for example, repeated blood sampling on the same individual, was incorporated as random effect and all analyses were controlled for age, body mass index, and fT4 concentrations. The effect of LT4 treatment on [3,5-T2] was tested using a Friedman ANOVA. Correction for multiple testing was done using the Benjamini–Hochberg procedure, controlling the false discovery rate at 5%. Multifluid data were integrated using a Gaussian graphical model (GGM; Supplementary Data) (38). Statistical analyses were carried out using SAS version 9.4 (SAS statistical software, version 9.4; SAS Institute, Inc., NC) and R 3.3.2 (version 3.3.2; R Foundation for statistical computing, Vienna, Austria).

## Results

**FIG. 1.**
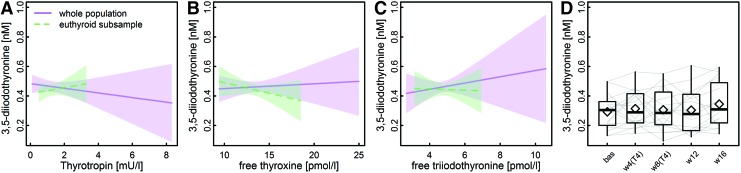
**(A–C)** Predicted means from linear regression analyses with thyroid function parameters as exposure and [3,5-T2] concentration as outcome in SHIP, either using the whole study population (solid) or a euthyroid subsample (dashed). **(D)** Boxplots indicate time courses of [3,5-T2] measurements during an LT4 challenge for 8 weeks among 16 healthy male volunteers [bas—baseline; w4(T4)/w8(T4)—four/eight weeks on 250 μg/day LT4; w12/w16–four/eight weeks after the LT4 application was finished]. Gray lines indicate individual time courses for the 16 volunteers. For none of the tested relationships, significant associations with [3,5-T2] could be detected. 3,5-T2, 3,5-diiodothyronine; LT4, levothyroxine. Color images are available online.

Characteristics of the study sample are summarized in Table 1. Concerning sex-specific differences, TSH and fT4 concentrations were slightly but significantly lower among men. As reported previously (27), no sex-specific difference in [3,5-T2] became apparent. Of special note, none of the common thyroid function parameters was significantly associated with [3,5-T2] (Fig. 1) and, moreover, [3,5-T2] was also unaltered during experimental LT4-induced thyrotoxicosis (Fig. 1).

**Table 1.** General Characteristics of the Study Population

Characteristics	Men (*N* = 370)	Women (*N* = 485)	p^a^
Age, years	50.0 (39.0; 60.8)	51.0 (41.0; 61.0)	0.91
Smoking, %			
Never	31.8	51.5	<0.01
Former	43.7	27.8	
Current	24.3	20.6	
Physical activity, %			
>1 hour/week	27.8	25.4	0.46
<1 hour/week	72.2	74.6	
Waist circumference	94.0 (86.5; 102.3)	81.0 (74.0; 89.7)	<0.01
Thyrotropin, mU/L	1.12 (0.78; 1.50)	1.23 (0.83; 1.78)	0.01
fT4, pmol/L	13.1 (12.2; 14.2)	13.5 (12.5; 14.7)	<0.01
fT3, pmol/L	4.97 (4.60; 5.31)	4.53 (4.23; 4.93)	<0.01
3,5-T2, nM	0.25 (0.20; 0.39)	0.25 (0.20; 0.37)	0.94

Data are expressed as median (25th; 75th percentile).

^a^ Wilcoxon rank-sum test for continuous and *χ*^2^-test for categorical data were used for comparison.

3,5-T2, 3,5-diiodothyronine; eGFR, estimated glomerular filtration rate; fT3, free triiodothyronine; fT4, free thyroxine.

### TH-independent association of [3,5-T2] with plasma and urine metabolites

**FIG. 2.**
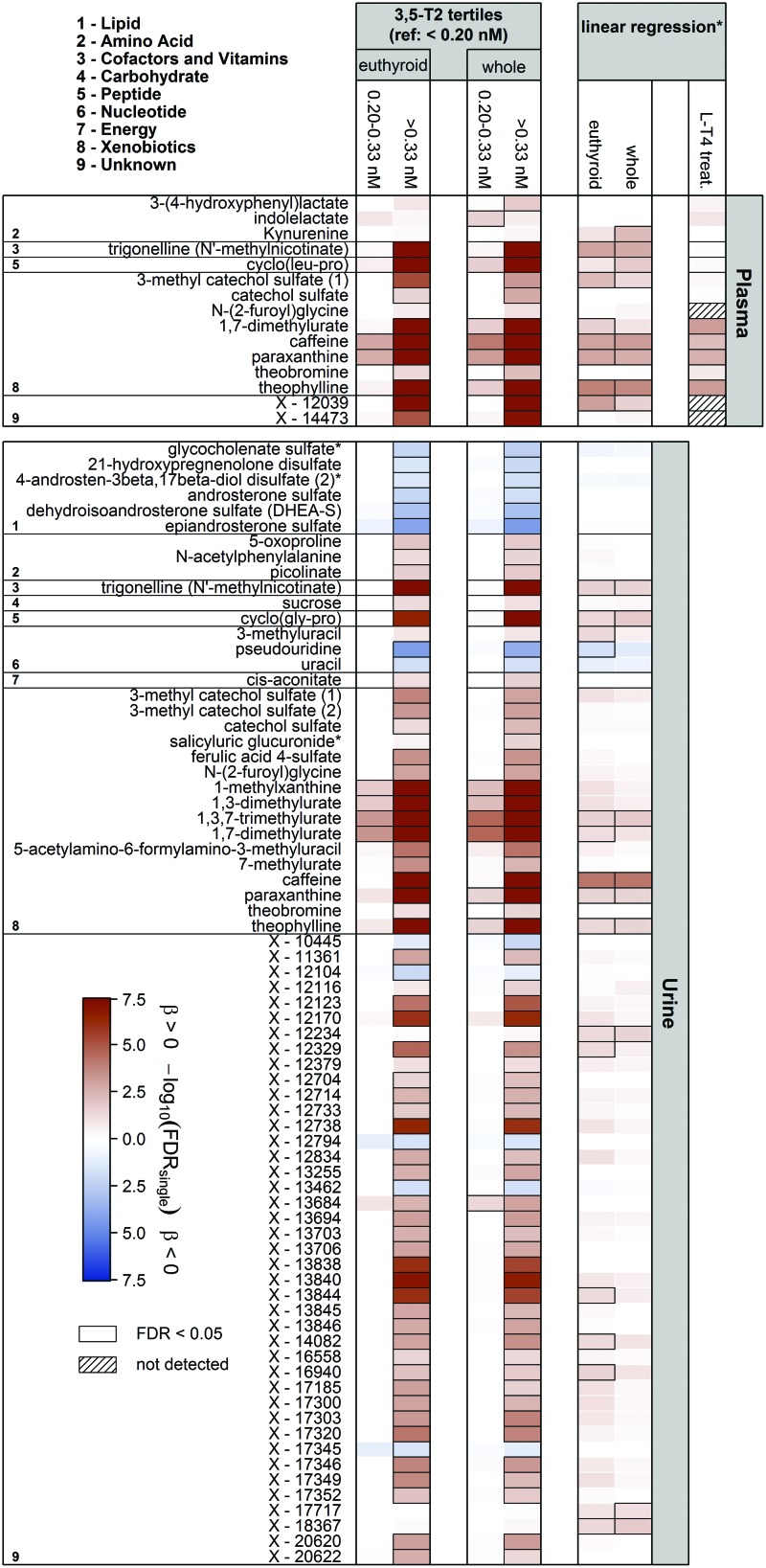
Heat map based on corrected *p*-values (controlling the FDR at 5%) from linear regression analyses using either tertiles of 3,5-diiodothyronine (3,5-T2; <0.20 nM, 0.20–0.33 nM, >0.33 nM) or log(3,5-T2) concentrations as exposure and plasma and urine metabolites as outcome. Models were adjusted for age, sex, waist circumference, time of blood sampling, and thyrotropin and fT4 concentrations. Columns indicate the effect comparing with the lowest 3,5-T2 group or results from linear regression using log(3,5-T2). Orange and blue shadings indicate positive and inverse associations, respectively. Thick frames indicate significant (FDR <0.05) associations. Analyses were performed on the whole sample as well as a euthyroid subsample. LT4 treat = associations between 3,5-T2 and named metabolites in an LT4 challenge; models were adjusted for age, sex, BMI, and fT4 concentrations. Corresponding estimates and FDR values are given in Table 2. *Linear regression analyses were only performed including participants with 3,5-T2 values above 0.20 nM. FDR, false discovery rate; fT4, free thyroxine. Color images are available online.

In total, [3,5-T2] was significantly associated with the concentrations of 15 and 73 metabolites in plasma and urine, respectively (Fig. 2). Notably, most of the associations became apparent in the comparison between the groups of low (<0.20 nM) versus high (>0.30 nM) [3,5-T2]. Exclusively positive associations were detected in plasma, including those with 3-(4-hydroxyphenyl)lactate, the tryptophan metabolites indole lactate and kynurenine, trigonelline, and the dipeptide cyclo(leu-pro).

**Table 2.** Estimates for Significantly Associated Metabolites That Are Also Available in the Thyrotoxicosis Sample

Metabolite	SHIP-TREND (*n* = 856)												Thyrotoxicosis study (*N* = 16 × 5 time points)	
	Spearman's correlation 3,5-T2			3,5-T2 groups (ref.: <0.20 nM)						log(3,5-T2) (>0.20 nM)			log(3,5-T2) (>0.20 nM)	
				0.20–0.33 nM			>0.33 nM							
	N	Rho	p	N	β*^a^*[95% CI]	FDR	N	β*^a^*[95% CI]	FDR	N	β*^a^*[95% CI]	FDR	β*^b^*[95% CI]	FDR
Caffeine	836	0.41	2.73E-35	257	0.42 [0.27–0.57]	5.44E-05	267	1.01 [0.86–1.16]	1.24E-33	538	0.38 [0.23–0.53]	4.23E-04	0.63 [0.41–0.85]	4.61E-03
Paraxanthine	830	0.39	1.80E-31	257	0.39 [0.23–0.54]	4.62E-04	268	0.85 [0.7–1.01]	1.14E-23	539	0.34 [0.19–0.5]	1.34E-03	0.57 [0.4–0.74]	1.63E-03
Theobromine	833	0.12	4.81E-04	255	0.11 [−0.06 to 0.27]	8.34E-01	268	0.33 [0.17–0.49]	4.98E-03	537	0.03 [−0.13 to 0.19]	9.69E-01	0.28 [0.1–0.47]	1.24E-01
Theophylline	754	0.38	6.54E-27	243	0.34 [0.17–0.5]	1.31E-02	250	0.92 [0.75–1.08]	6.84E-24	505	0.43 [0.27–0.59]	1.49E-04	0.5 [0.38–0.62]	4.94E-04
1,7-Dimethylurate	616	0.32	8.88E-16	194	0.38 [0.19–0.57]	1.31E-02	226	0.81 [0.63–0.99]	3.34E-15	428	0.3 [0.12–0.48]	7.32E-02	0.51 [0.38–0.64]	4.94E-04
3-(4-Hydroxyphenyl)lactate	791	0.11	1.31E-03	241	0.18 [0.03–0.33]	5.46E-01	259	0.27 [0.13–0.42]	1.02E-02	512	0.05 [−0.08 to 0.19]	9.20E-01	0.04 [0.01–0.08]	2.19E-01
Trigonelline	785	0.29	3.36E-16	241	0.25 [0.08–0.41]	3.01E-01	257	0.69 [0.53–0.85]	3.31E-14	510	0.39 [0.23–0.56]	1.10E-03	−0.03 [−0.15 to 0.08]	7.76E-01
Cyclo(leu-pro)	794	0.27	1.20E-14	241	0.33 [0.16–0.49]	1.31E-02	263	0.67 [0.51–0.83]	5.24E-14	517	0.31 [0.15–0.46]	1.04E-02	0.07 [−0.03 to 0.16]	4.88E-01
3-Methyl catechol sulfate (1)	523	0.19	7.50E-06	160	0.07 [−0.14 to 0.28]	9.13E-01	180	0.47 [0.27–0.67]	3.89E-04	346	0.38 [0.17–0.59]	2.90E-02	0.21 [−0.01 to 0.44]	3.29E-01
Catechol sulfate	832	0.14	3.69E-05	255	0.14 [−0.02 to 0.3]	7.48E-01	266	0.35 [0.19–0.51]	1.00E-03	535	0.13 [−0.02 to 0.29]	6.59E-01	0.05 [−0.03 to 0.13]	5.15E-01

^a^ Linear regression models adjusted for age, sex, waist circumference, blood sampling time, as well as serum concentrations of fT4 and thyrotropin, %-change compared with the lowest group could be achieved by exponentiation of the beta-estimate to the base of 2 and subtraction of 1 multiplied by 100 [(2^β^−1)×100].

^b^ Mixed effects linear regression model adjusted for age, body mass index, and fT4 concentrations.

CI, confidence interval; FDR, false discovery rate; ref., reference group; SHIP-TREND, Study of Health in Pomerania-TREND.

**FIG. 3.**
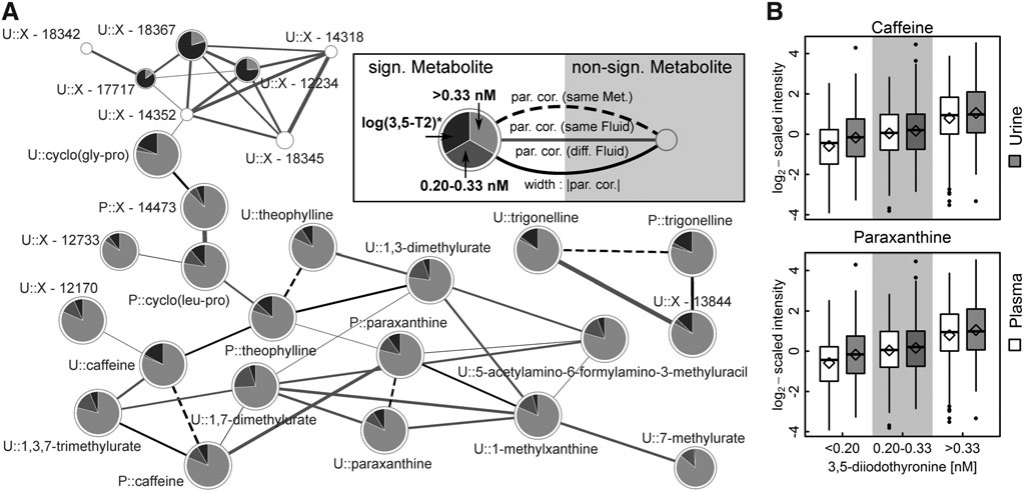
**(A)**Subnetwork of the derived metabolic network (estimated by Gaussian graphical modeling) emphasizing caffeine and related compounds. On each node, the results from linear regression analysis for 3,5-T2 concentration either as categorical (effect compared with the lowest group, that is, <0.20 nM; dark gray: 0.20–0.33 nM and light gray: >0.33 nM) or continuous variable (*considering participants with [3,5-T2] >0.20 nM; black) as portion of the association strength are given as −log10 (FDR-value). Significant results in at least one of these with an FDR value below 5% are depicted as pie charts. Node sizes are inversely proportional to the lowest FDR-value. The prefixes P and U denote plasma and urine metabolites, respectively. Edges represent significant partial correlations (par. cor.) between metabolites. **(B)** Boxplots of plasma (blank) or urine (gray) levels of caffeine and paraxanthine across groups of [3,5-T2]. Diamonds indicate mean levels.

Strikingly, the strongest [3,5-T2] associations were found with metabolites related to caffeine metabolism, namely caffeine, paraxanthine, theobromine, and theophylline (Fig. 2 and Table 2). For instance, the average caffeine levels in the middle and the high [3,5-T2] groups were about 33% and 100% increased, respectively, compared with the lowest [3,5-T2] group. Similarly, paraxanthine and theophylline levels in plasma increased with 3,5-T2 concentrations in these groups (Table 2). These findings were also reflected in the urine metabolome where additionally significant positive [3,5-T2] associations with the levels of 1,3,7-trimethylurate, 1,3-dimethylurate, and 1,7-dimethylurate were revealed (Fig. 2). Visual inspection of the derived metabolic network (GGM) strongly supported the close interrelationship between those metabolites (Fig. 3), which further comprised 5-acetylamino-6-formylamino-3-methyluracil, the dipeptides cyclo(leu-pro) in plasma and cyclo(gly-pro) in urine, as well as several unknown compounds.

A second cluster of significantly positively [3,5-T2] associated metabolites in urine comprised N-(2-furoyl)glycine, ferulic acid 4-sulfate, as well as several unknown compounds. Further positive associations were noted with the urine levels of 5-oxoproline, N-acetyl phenylalanine, picolinate, sucrose, 3-methyluracil, cis-aconitate, as well as the benzoate metabolites 3-methyl catechol sulfate and catechol sulfate. Notably, the [3,5-T2] association with urinary hippurate barely missed the corrected statistical significance. Inverse associations were limited to urine levels of sulfated compounds belonging to steroids, for example, dehydroisoandrosterone sulfate or 21-hydroxypregnenolone sulfate (Fig. 2), pseudouridine, uracil, and five unknown compounds.

When the analyses were restricted to a euthyroid subsample (Materials and Methods section), almost all associations remained significant. This included all primary catabolites of caffeine as well as other surrogates of coffee consumption such as trigonelline. The loss of few associations, including plasma kynurenine, indole lactate, and 3-(4-hydroxyphenyl)lactate, as well as five unknown compounds in urine, was most likely due to the resulting reduction in statistical power rather than to a specific effect of thyroid function since these associations initially reached statistical significance only tightly below the applied threshold.

### Further adjustment for plasma caffeine

To test whether the associations between [3,5-T2] and metabolite levels were of distinct origin, we further adjusted the linear models for the concentration of plasma caffeine as the [3,5-T2] association with this metabolite was the strongest in the present study. In consequence, all significant associations with [3,5-T2] were lost, with the sole exception of plasma kynurenine levels in case of using [3,5-T2] as continuous exposure. Similarly, if our previous analyses (28) were repeated with additional adjustment for plasma caffeine, significant associations were no longer observed either.

### Replication of the main findings in a human model of experimental LT4-induced thyrotoxicosis

Even under severe thyrotoxic conditions (mean TSH <0.02 mU/L and mean fT4 > 28.6 pmol/L) after eight weeks of LT4 treatment, no significant alterations in [3,5-T2] became obvious (*p* = 0.68; Fig. 1). Strikingly, the strong positive associations between [3,5-T2] and central caffeine metabolites, that is, caffeine, paraxanthine, 1,7-dimethylurate, and theophylline were replicated in this rather small study cohort (*n* = 16) (Fig. 2 and Table 2).

## Discussion

The primary aim of the present study was the detection of clues for metabolic relevance of 3,5-T2 in humans by performing association analyses between [3,5-T2] and the concentrations of plasma and urine metabolites, using available comprehensive metabolome data in a relatively large population-based and a smaller interventional human thyrotoxicosis sample. Particularly, we searched for possible thyroid function-independent effects that appeared plausible, because previous studies did not reveal obvious associations between the concentrations of 3,5-T2 and those of T4 or T3 (27,28,39–41). These were expected as it was hypothesized that classical TH might represent direct precursors for 5′-deiodinase-mediated 3,5-T2 formation. Furthermore, 3,5-T2 was not found to be produced by the thyroid gland itself (42).

The results presented here indicate a clear link between [3,5-T2] and coffee metabolism as reflected by the associated metabolites. This relationship was also observed in the experimental thyrotoxicosis model, confirming that [3,5-T2] is not primarily determined by circulating TH concentrations and therefore does not reflect the actual thyroid functional state.

### The metabolomic fingerprint of 3,5-T2 is not thyroid function dependent

Despite numerous results from animal studies revealing complementary effects of 3,5-T2 to classical TH, there is still ongoing debate about the major site(s) and mode of 3,5-T2 synthesis and its physiological relevance in humans. Detection of 3,5-T2 in the circulation of thyroidectomized patients treated with LT4 demonstrated extrathyroidal conversion of classical TH to 3,5-T2 (27). However, our results strongly argue against a simple thyroid function- and TH dose-dependent [3,5-T2] production. First, no significant associations with circulating TH were detected as expected in the case of constant rate conversion of TH to 3,5-T2. Strikingly, in the present study, these were missing in the population-based cohort as well as the thyrotoxic individuals. While these findings partially contrast with our previous results on the interrelationship of TSH with [3,5-T2] in human serum, they are in line with negative reports on a simple TH dependency of [3,5-T2] (22,27).

Second, the metabolomic 3,5-T2 fingerprint, especially the main signature of caffeine-related metabolites, was quite different from those described for thyroid diseases or circulating TH (36,43–46). The fingerprint of TH in plasma is dominated by lipid species, γ-glutamyl dipeptides, and shifts in amino acid proportions (47). Merely for the urine metabolome, a small overlap between the fingerprints of 3,5-T2 and classical TH was observed, as hippurate and trigonelline were also associated with the TSH and fT4 serum concentrations, respectively, in population-based studies (43,48). While several animal studies (14–16,19) reported dose-dependent thyromimetic effects of 3,5-T2 on the HPT-axis and the heart (4), such supraphysiological concentrations in the circulation required to exert these adverse effects were most probably not reached in our study samples. Nevertheless, we cannot rule out that endogenous 3,5-T2 affects the HPT-axis, but the independence of the metabolomic 3,5-T2 fingerprint from TSH and fT4 concentrations in our two study cohorts strongly argues for a metabolic fate of 3,5-T2 distinct from that of the classical TH.

### 3,5-T2 is interrelated with the metabolism of coffee ingredients

The strongest associations with [3,5-T2] were detected for caffeine and its direct catabolites (49), for example, paraxanthine, theophylline, and 1-methylxanthine, in plasma as well as urine. Furthermore, numerous metabolites related to coffee consumption were positively associated with [3,5-T2] in plasma and/or urine, including trigonelline present in green coffee that passes through the body without undergoing substantial phase II metabolism (49), N-(2-furoyl)glycine metabolized from furan derivatives present in coffee products (50), and the proline-based diketopiperazine cyclo(leu-pro) generated in the roasting process, which contributes to the bitter taste of coffee (51). These associations were also observed under the conditions of experimental thyrotoxicosis. While these results strongly argue for a causative relationship between coffee consumption and [3,5-T2], this interpretation, because based exclusively on association findings, has nevertheless to be treated with appropriate caution.

### Known effects of coffee on hepatic detoxification systems

The central question arising from these results concerns the physiological mechanisms underlying the observed associations. Potential explanations outlined below are based on the fact that the liver, the most important detoxification organ of the human body, is strongly affected by coffee.

Transcriptional induction by coffee was demonstrated for the regulon controlled by the transcription factor aryl hydrocarbon receptor (AHR) using hepatic cell lines and livers of a humanized murine model (52). The AHR is a nuclear receptor that after activation by multiple endogenous and exogenous ligands (53) translocates to the nucleus, dimerizes with the AHR nuclear translocator protein, subsequently binds to xenobiotic response elements, and induces expression of a large regulon. Hepatic murine transcriptome analyses identified several 100 AHR target genes (54), where many of the encoded proteins are involved in xenobiotic metabolism (53).

Among the AHR target genes in humans as well as mice is *NFE2L2* encoding NRF2, the master regulator of another major cellular defense system against oxidative and other cytotoxic stress (55). After nuclear translocation, NRF2 binds to antioxidant response element enhancer sequences located in promoters of many genes encoding proteins necessary for electrophile detoxification (56). Consequently, AHR regulon induction also causes transcriptional activation of the NRF2 regulon comprising more than 200 genes as indicated by transcriptome analyses, including genes encoding proteins involved in protection against oxidative stress and multidrug response transporters (56,57).

Thus, extensive transcriptional reprogramming of the liver resulting in altered enzyme and transporter expression appears to be caused by (chronic) coffee consumption. Of note, as transcriptional upregulation of AHR target genes is also mediated by decaffeinated coffee, other coffee ingredients than caffeine are responsible for initial activation of the AHR regulon [44].

### Beneficial health effects of coffee consumption

In summary, the strong hepatic activation of the cytoprotective and genoprotective AHR and NRF2 systems by coffee suggests to cause increased detoxification and elimination of xenobiotics, oxidative intermediates, and reactive oxygen species generated by phase I metabolism is in line with the beneficial health effects of moderate coffee consumption that were observed in many large epidemiological studies and are today beyond controversy. For example, a multinational cohort study analyzing data of a sample of 521,330 individuals detected a reduced risk for death from various causes associated with coffee consumption (58). In particular, coffee consumption is associated with a decreased risk of several chronic diseases, including type 2 diabetes (T2D), cardiovascular disease (CVD), and cancer, as well as neurodegenerative conditions such as Parkinson's disease (49).

Mechanistically, coffee-mediated activation of the AHR and NRF2 regulons enhances the general detoxification capacity of the liver and in particular the potential in neutralization of reactive oxygen species. Upregulation of the synthesis of the key enzyme of glutathione (GSH) synthesis, hepatic γ-glutamylcysteine synthetase, the most important site of GSH synthesis and storage (59), explains increased GSH concentrations in plasma (60,61) and colorectal mucosa (60) observed after daily coffee consumption. Indeed, a systemic and organ-specific improvement of the detoxification capacity might explain the reduced cancer risk associated with coffee consumption, as well as the significant inverse association between moderate coffee drinking and CVD risk (62).

In case of T2D, a lower risk of coffee drinkers to develop the disease was demonstrated in several studies (63–65). As chronical oxidative stress triggers the development of T2D (66), coffee-mediated activation of corresponding protection systems might contribute to this observed risk reduction. However, another well-defined effect of coffee might be even more important, namely its impressive potential to reduce the hepatic fat content: there is a strong epidemiological association between T2D and hepatic steatosis, and several common models on the pathogenesis of insulin resistance and, subsequently, T2D include the development of a fatty liver as key event [e.g., (67)]. Notably, epidemiological evidence also indicates that coffee consumption reduces the hepatic fat content (68,69), as well as liver enzyme serum activities (58). Therefore, hepatic fat reduction is suggested to be a crucial factor in lowering T2D risk by coffee consumption.

The underlying mechanism is, however, not yet fully understood. Although stimulation of intrahepatic lipolysis and fatty acid oxidation *via* caffeine-induced flux through an autophagy/lysosomal pathway was demonstrated in a murine model (70), human intervention studies revealed only small effects on weight reduction and fat metabolism (71). Furthermore, of particular importance, the inverse relationships between coffee consumption and T2D reported in Refs. (72) and (65) were observed for caffeinated as well as decaffeinated coffee.

### Effects of TH and their derivatives on liver fat catabolism

Of particular relevance in the context of the present study, TH are known to reduce the hepatic triglyceride content, *via* stimulation of lipophagy of intracellular lipid droplets (73), as well as transcriptional induction of *CPT1* encoding carnitine palmitoyltransferase-I, a direct target of TH-activated TH receptor α and β (TR) (74). Induction of hepatic *CPT1* expression causes intensified mitochondrial β-oxidation and, consequently, fat burning. In addition, activation of the citrate cycle is ensured by TH-mediated transcriptional upregulation of *IDH3A* encoding the α-subunit of mitochondrial NAD^+^-dependent isocitrate dehydrogenase 3 (75–77). Therefore, TH agonists could principally represent promising drugs for the treatment of hepatic steatosis and obesity-related diseases, but harmful effects of the studied agonists on heart, bone, and muscle have, so far, prevented their use as therapeutic compounds (18,78).

Of special interest in relation to the present work, the antihepatosteatotic potential of TH as well as its unwanted effects in other organs applies not only to the classical TH but also for their metabolite 3,5-T2 (4–13,18), as outlined in the Introduction section. In particular, the characteristics of 3,5-T2 motivated our association study to get further information on its putative physiological role.

### Hypothetical explanations for the association between [3,5-T2] and coffee metabolites

Considering the described interrelationships between T2D, hepatic steatosis, the protective effects of coffee consumption on these disease entities, as well as its strong impact on hepatic metabolism *via* activation of the AHR-NFR2 system, and the significant potential of classical TH and 3,5-T2 to reduce liver fat and induce drug-metabolizing enzymes (13), one might hypothesize that the observed association between [3,5-T2] and coffee metabolites reflects the intrahepatic physiological state, thereby indicating coffee-induced 3,5-T2 production and accumulation with proportional excretion into the circulation.

According to this model, one or more coffee compounds aside from caffeine may induce the AHR-NRF2 axis, where the subsequent extensive hepatic gene expression reprogramming, by an unidentified molecular mechanism, mediates an increase in the hepatic 3,5-T2 concentrations. This could be accomplished either *via* stimulated generation, for example, by enhanced deiodination of TH or reduced catabolism of 3,5-T2. By activation of the liver-dominating TRβ and transcriptional upregulation of the corresponding regulon, the increased intrahepatic 3,5-T2 concentrations could subsequently stimulate the hepatic fat catabolism mechanisms. The resulting reduction in hepatic fat content might finally reduce the risk for hepatic steatosis and secondary disorders, a concept that is consistent with the reported epidemiological findings.

Of course, other explanations are conceivable: for instance, the coffee-mediated activation of the AHR-NFR2 system could induce expression of one or more hepatic 3,5-T2 exporter proteins, which would also explain the observed associations. However, the possibility of a putative key role of endogenous 3,5-T2 in hepatic metabolism should encourage further specific animal model-based experiments to clarify the mechanism explaining the observed association between [3,5-T2] and coffee metabolites. The fact that this finding represents the first clear association between [3,5-T2] and a physiologically relevant trait in a larger human study sample emphasizes the potential of nontargeted OMIC studies to increase the understanding of complex physiological relationships.

### Strengths and limitations

A clear strength of the present study consists in the availability of both large-scale metabolome data and classical measures of thyroid function that allows to derive information on the specific metabolic fate of 3,5-T2, where the endogenous role of the latter in humans is still unclear. Furthermore, the observed associations between [3,5-T2] and coffee metabolites do not rely on self-reported coffee consumption as in many epidemiological studies, but on measured concentrations of specific ingredients of coffee in plasma and urine samples. However, given that these data are based on associations, the results are of observational nature and required in part transformation and statistical evaluation of the comprehensive metabolomic data sets; therefore, the data do not allow to assume a causal relationship. For these reasons, appropriate animal model-based intervention studies or a coffee-ingestion trial in humans is required to test these hypotheses.

In conclusion, using state-of-the-art metabolic profiling to analyze plasma and urine samples of a larger human study population, we were able to detect a novel connection between serum 3,5-T2 concentrations and coffee metabolism. The hypothetical mechanisms, that is, that coffee-induced intrahepatic accumulation of 3,5-T2 initiates liver fat reduction *via* activation of TR-dependent transcriptional regulation, have to be tested in appropriate experimental model systems. In addition, we were able to demonstrate that serum 3,5-T2 concentrations are widely independent from the classical parameters of thyroid function, that is, TSH, fT4, and fT3.

## Supplementary Material

Supplemental data
