# Supplementary material for: A Thyroid Hormone-Independent Molecular Fingerprint of 3,5-Diiodothyronine Suggests a Strong Relationship with Coffee Metabolism in Humans
Source: Thyroid. 2019 Dec 16;29(12):1743–54. doi: 10.1089/thy.2018.0549 (PMC6918876; doi:10.1089/thy.2018.0549)
Supplement: Supplemental data [file Supp_Data.pdf]

Supplementary Data

Methods

Untargeted liquid chromatography with tandem mass spectrometry profiling

Metabolomic measurements. Nontargeted metabolomic analysis for metabolic profiling was conducted at the Genome Analysis Center, Helmholtz Zentrum München. Two separate liquid chromatography with tandem mass spectrometry (LC-MS/MS) analytical methods as previously published, that is, in positive and in negative ionization modes, were used to detect a broad metabolite panel (S1).

In this study, samples were divided into two sets according to the biological matrices of the samples, that is, plasma and urine. On the day of extraction, samples were thawed on ice. A 100 μL of the sample was pipetted into a 2 mL 96-well plate. In addition to study samples, a human pooled reference plasma sample (Seralab, West Sussex, United Kingdom) and another pooled reference matrix of each sample set (Seralab) were extracted and placed in 1 and 6 wells, respectively, of the 96-well plate. These samples served as technical replicates throughout the data set to assess process variability. Beside those samples, 100 μL of water was extracted as samples and placed in 6 wells of the 96-well plate to serve as process blanks. Protein was precipitated and the metabolites were extracted with 475 μL methanol, containing four recovery standards to monitor the extraction efficiency. After centrifugation, the supernatant was split into four aliquots of 100 μL each onto two 96-well microplates. The first two aliquots were used for LC-MS/MS analysis in positive and negative electrospray ionization modes. Two further aliquots were kept as a reserve. The extracts were dried on a TurboVap 96 (Zymark, Sotax, Lörrach, Germany).

Before LC-MS/MS in positive ion mode, the samples were reconstituted with 0.1% formic acid (50 μL for plasma and 100 μL for urine). Whereas samples analyzed in negative ion mode were reconstituted with 6.5 mM ammonium bicarbonate (50 μL for plasma and 100 μL for urine), pH 8.0. Reconstitution solvents for both ionization modes contained internal standards that allowed monitoring of instrument performance and also served as retention reference markers. To minimize human error, liquid handling was performed on a Hamilton Microlab STAR robot (Hamilton Bonaduz AG, Bonaduz, Switzerland).

LC-MS/MS analysis was performed on a linear ion trap LTQ XL mass spectrometer (Thermo Fisher Scientific GmbH, Dreieich, Germany) coupled with a Waters Acquity UPLC system (Waters GmbH, Eschborn, Germany). Two separate columns (2.1 × 100 mm Waters BEH C18, 1.7 μm particle-size) were used for acidic (solvent A: 0.1% formic acid in water, solvent B: 0.1% formic acid in methanol) and/or for basic (A: 6.5 mM ammonium bicarbonate, pH 8.0, B: 6.5 mM ammonium bicarbonate in 95% methanol) mobile-phase conditions, optimized for positive and negative electrospray ionization, respectively. After injection of the sample extracts, the columns were developed in a gradient of 99.5% A to 98% B over an 11-minute run time at 350 μLl/min flow rate. The eluant flow was directly run through the ESI source of the LTQ XL mass spectrometer. The mass spectrometer analysis alternated between mass spectrometry (MS) and data-dependent MS/MS scans using dynamic exclusion and the scan range was from 80 to 1000 m/z.

Metabolites were identified by Metabolon, Inc., from the LC-MS/MS data by automated multiparametric comparison with a proprietary library, containing retention times, m/z ratios, and related adduct/fragment spectra (S2). Identification criteria for the detected metabolites are described in Evans *et al.* (S1). Quality control methods and normalization of metabolite levels are explained below.

Metabolomic measurements: quality control and normalization of metabolite levels. To correct for daily variations of platform performance, the raw ion count of each metabolite was rescaled by the respective median value of the run day. Valid estimation of the median was ensured by keeping only metabolites with at least three measured values on more than half of the run days. This procedure resulted in 475 and 558 metabolites for plasma and urine, respectively, available for the present analysis. Two hundred sixty-three metabolites were measured in both biofluids.

We chose probabilistic quotient normalization (PQN) (S3) to account for diurnal variation of urine samples, since this procedure was shown to be superior to the common creatinine scaling. For this purpose, we calculated a mean pseudospectrum depending on metabolites with measurements for all participants (131 urine metabolites). Subsequently, we calculated a dilution factor as the median quotient between the reference spectrum and each sample. Of note, urine creatinine and the estimated dilution factor were highly correlated ($r = 0.91$, $p < 0.001$) within the present study sample. Afterward, all metabolite levels were $\log_2$-transformed.

Separately for plasma and urine samples, we performed multivariate outlier detection using an algorithm proposed by Filzmoser *et al.* (S4) as implemented in the *pcout* function within the R package *mvoutlier*. The algorithm provides an outlier score for each sample based on a weighted combination of location and scatter estimations using principal component analysis and the Mahalanobis distance on a robustly scaled data matrix. The default parameters were used for the identification process, except that the critical value for the location outliers was set to 4, as it corresponds to a 4 standard deviation (SD) exclusion criteria. The minimum score was used as cutoff for outlier identification. As a result, 13 and 8 samples from plasma and urine were excluded, respectively.

Targeted LC-MS/MS profiling

Metabolomic measurements. Targeted metabolomic profiling of the serum samples was performed using the AbsoluteIDQ p180 Kit (BIOCRATES LifeSciences AG, Innsbruck, Austria, online Supplementary Methods section). Ten microliter aliquots of each plasma sample were processed as recommended by the manufacturer. The fully automated assay combined flow injection analysis (FIA) and LC-MS/MS

selective detection using MRM pairs and quantifies up to 188 metabolites from 5 different compound classes. Via FIA acyl carnitines, phospho- and sphingolipids were measured in positive ionization mode and the sum of hexoses in negative ionization mode. With an LC-MS/MS analytical method, under the use of an Agilent C18 column, amino acids and biogenic amines were detected. MS analyses were performed on an AB SCIEX 5500 QTrap™ mass spectrometer (AB SCIEX, Darmstadt, Germany) with electrospray ionization combined with an HPLC system (Agilent 1260 Infinity Binary LC, Santa Clara, United States), including a degasser unit, column oven, autosampler, and a binary pump. Internal standards (isotope labeled) are partially integrated in the kit plate for metabolite quantification. After the measurement, a preprocessing step includes peak integration and concentration determination from calibration curves, with Analyst software (version 1.5.1; AB Sciex, Darmstadt, Germany), data were uploaded into Biocrates MetIDQ software (part of the kit), and the metabolite concentrations were automatically calculated with it.

Metabolomic measurements: quality control and normalization of metabolite levels. To account for between-plate variation, a solely sample-dependent normalization was performed. To this end, for each plate, the measured concentrations of the metabolites were divided by the median concentration leading to equal median values for each metabolite on each plate. Subsequently, the median of the plate medians was calculated to reset to the original scale (μM concentrations). No obvious pattern in missing values along the measurement period became obvious. However, only metabolites with at least 20% valid observations were included in the final data sets, resulting in 183 being used for subsequent analysis. Principle component analyses were performed to detect multivariate outliers. These were defined as samples deviating more than three times the SD from the mean Mahalanobis distance based on the first ten principal components. As a result, four samples were excluded. Finally, metabolite levels were $\log_2$-transformed.

^{1}H-NMR profiling

Metabolomic measurements. Prior analysis urine specimens were stored about five years at –80°C. After thawing, urine specimens were centrifuged for 5 min at 3000 *g* and the supernatant was used for spectroscopic analysis. To this purpose, 450 μL of urine was mixed with 50 μL phosphate buffer to stabilize the urinary pH at 7.0 (±0.35). The phosphate buffer was prepared with D2O and contained sodium 3-trimethylsilyl-(2,2,3,3-D4)-1-propionate (TSP) as reference. Spectra were recorded at the University Medicine Greifswald, Germany, on a Bruker DRX-400 NMR spectrometer (Bruker BioSpin GmbH, Rheinstetten, Germany) operating at 1H frequency of 400.13 MHz and equipped with a 4-mm selective inverse flow probe (FISEI, 120 μL active volume) with z-gradient. Specimens were automatically delivered to the spectrometer via flow injection. The acquisition temperature was set to 300°K.

A standard one-dimensional 1H-NMR pulse sequence with suppression of the water peak (NOESYPREAST) was used: RD—P(90°)–4 microseconds—P(90°)—t_m—P(90°)—acquisition of the free induction decay (FID). The nonselective 90° hard pulse P(90°) was adjusted to 9.4 microseconds. The relaxation delay (RD), the mixing time (t_m), and the acquisition time were set to 4 seconds, 100 milliseconds, and 3.96 seconds, respectively, resulting in a total recycle time of ~8.0 seconds. Low-power continuous-wave irradiation on the water resonance at a field strength of ~25 Hz was applied during RD and t_m for presaturation. After application of 4 dummy scans, 32 FIDs were collected into 32,768 (32K) complex data points using a spectral width of 20.689 parts per million (ppm). FIDs were multiplied with an exponential function corresponding to a line broadening of 0.3 Hz before Fourier-transformation. Spectra were manually phase and baseline corrected and automatically referenced to the internal standard (TSP—0.0 ppm) within TopSpin 1.3 (Bruker BioSpin).

Metabolomic measurements: quality control and normalization of metabolite levels. The Fourier-transformed and baseline-corrected NMR spectra were manually annotated by spectral pattern matching using Chenomx NMR Suite 6.1 (Chenomx, Inc., Edmonton, Alberta, Canada) to deduce absolute urinary concentrations of 56 metabolites; subsequently, the NMR data were reduced to these metabolites. Similar to MS measurements, urinary dilution was accounted for by PQN normalization. Normalized metabolite levels were once more $\log_2$-transformed.

Additional data processing

Data integration. Most of the metabolites were unique to one of the applied techniques. However, 44 and 22 metabolites in plasma and urine, respectively, were overlapping with at least one other technique. With respect to plasma, following the grouping of metabolites in biochemical classes (i.e., lipids, amino acids, and carbohydrates), correlations of those metabolites measured on both platforms were computed with all members of the same biochemical class. Subsequently, the metabolite with the higher median correlation across all class members was kept for further analysis. Regarding the lower sensitivity of NMR compared with MS, in cases of duplicated measures, those obtained on the MS were kept for further analyses. In total, 613 plasma and 587 urine metabolites were used in the subsequent statistical analyses.

To avoid spurious results in linear regression analysis, univariate outliers for each metabolite were excluded whenever concentrations exceeded more than three standard deviations from the mean value.

Statistical analyses

Gaussian graphical model. To facilitate integration of multifluid data, we computed a Gaussian graphical model (GGM) because of its ability to reconstruct physiological dependencies purely in a data-driven manner (S5). This allowed us to evenly make assumptions about the biochemical context of up to now unknown compounds. Briefly, a GGM relies on full-order partial correlations, which means that a correlation between two metabolites only exists if it is independent from all remaining metabolites in the data set. Significant partial correlations after Bonferroni correction were visualized as network using Cytoscape 3.2.1. Since GGMs require a full data matrix, missing values were imputed using the k-nearest-neighbor imputing as implemented in the R-package *impute*. K was set to 10 and

only metabolites with less than 20% missing values were considered ($N = 872$). The final GGM comprised 785 nodes and 1065 edges.

References

S1. Evans AM, DeHaven CD, Barrett T, Mitchell M, Milgram E 2009 Integrated, nontargeted ultrahigh performance liquid chromatography/electrospray ionization tandem mass spectrometry platform for the identification and relative quantification of the small-molecule complement of biological systems. Anal Chem **81:**6656–6667.

S2. Lawton KA, Berger A, Mitchell M, Milgram KE, Evans AM, Guo L, Hanson RW, Kalhan SC, Ryals JA, Milburn MV 2008 Analysis of the adult human plasma metabolome. Pharmacogenomics **9:**383–397.

S3. Dieterle F, Ross A, Schlotterbeck G, Senn H 2006 Probabilistic quotient normalization as robust method to account for dilution of complex biological mixtures. Application in 1H NMR metabonomics. Anal Chem **78:**4281–4290.

S4. Filzmoser P, Maronna R, Werner M 2008 Outlier identification in high dimensions. Comput Stat Data Anal **52:**1694–1711.

S5. Krumsiek J, Suhre K, Illig T, Adamski J, Theis FJ 2011 Gaussian graphical modeling reconstructs pathway reactions from high-throughput metabolomics data. BMC Syst Biol **5:**21.
